# Post-operative radiation therapy to the surgical cavity with standard fractionation in patients with brain metastases

**DOI:** 10.1038/s41598-020-63158-6

**Published:** 2020-04-14

**Authors:** James D. Byrne, Thomas Botticello, Andrzej Niemierko, Helen A. Shih, Jay S. Loeffler, Kevin S. Oh

**Affiliations:** 1 Harvard Radiation Oncology Program, Harvard Medical School, Boston, MA USA; 2 Department of Radiation Oncology, Massachusetts General Hospital, Boston, MA USA

**Keywords:** CNS cancer, Metastasis

## Abstract

The paradigm for post-operative cavity radiation therapy has shifted to more targeted, less morbid approaches. Single-fraction or hypofractionated radiation therapy is a common approach to treating the postoperative cavity but is associated with a local failure rate 20–40%. We employed an alternative treatment strategy involving fractionated partial brain radiation therapy to the surgical cavity. Patients with brain metastases who underwent radiation treatment 30–42 Gy in 3 Gy/fraction regimens to surgical cavity were retrospectively identified. The 6-month and 12-month freedom from local failure rates were 97.0% and 88.2%. Three patients (7%) experienced local failure at 4, 6, and 22 months. Of these, the histologies were colorectal adenocarcinoma (N = 1) and breast adenocarcinoma (N = 2). The 6-month and 12-month freedom from distant brain metastases rates were 74.1% and 68.8%, respectively, and the 6-month and 12-month overall survival rates were 84.9% and 64.3% respectively. The median overall survival was 39 months, and there were no events of late radionecrosis. Fractionated partial brain irradiation to the surgical cavity of resected brain metastases results in low rates of local failure. This strategy represents an alternative to SRS and WBRT.

## Introduction

As patients are living longer due to improvements in systemic therapies, the incidence of brain metastases has been increasing^1,2^. Surgical resection of brain metastases is an important modality for management and has been shown to improve survival when compared to whole brain radiation therapy (WBRT) alone. However, local failure after resection approaches 60–70%^3^. Multiple randomized trials have demonstrated that the addition of post-operative whole brain radiation therapy is associated with improving both local and distant intracranial control^4–7^. The paradigm in post-operative radiation therapy to the cavity has significantly evolved over the past 10 years to more targeted and less morbid techniques, including stereotactic radiosurgery (SRS) and hypofractionated stereotactic radiotherapy (SRT)^8–13^.

Two recently published prospective trials have demonstrated a local failure rate of 20–40% when using single-fraction radiosurgery^8,9^. Consensus contouring guidelines suggest that the postoperative target should include the entire surgical tract and 5–10 mm extension along the dura, a volume that might be underestimated when using single-fraction SRS^10^. Paradoxically, lower conformality has been correlated with improved local control in patients treated with SRS to the resection cavity, which suggests that generous volumes may be beneficial^11^.

There is the major concern for side effects from SRS and SRT regimens, including radionecrosis, reduction in neurocognition, seizures, nausea, and headaches^14–17^. Alternatively, an approach that uses more standard fractionation may allow for more generous treatment volumes and widened therapeutic index between tumor and normal tissues. In this study, we evaluate the use of post-operative cavity radiation therapy with standard fractionation with respect to local control, distant intracranial control, and late radionecrosis.

## Methods and Materials

### Patient characteristics

This retrospective study was approved by the Partners Human Research Committee/Institutional Review Board. Partners Human Research Committee/Institutional Review Board waived the need for informed consent as part of their study approval. Forty-five consecutive patients with brain metastases underwent gross total resection during routine care and were treated with involved-field radiation treatment to a total dose of 30–42 Gy in 3 Gy/fractions at the Massachusetts General Hospital between April 2012 and September 2017.

### Treatment

**Figure 1:**
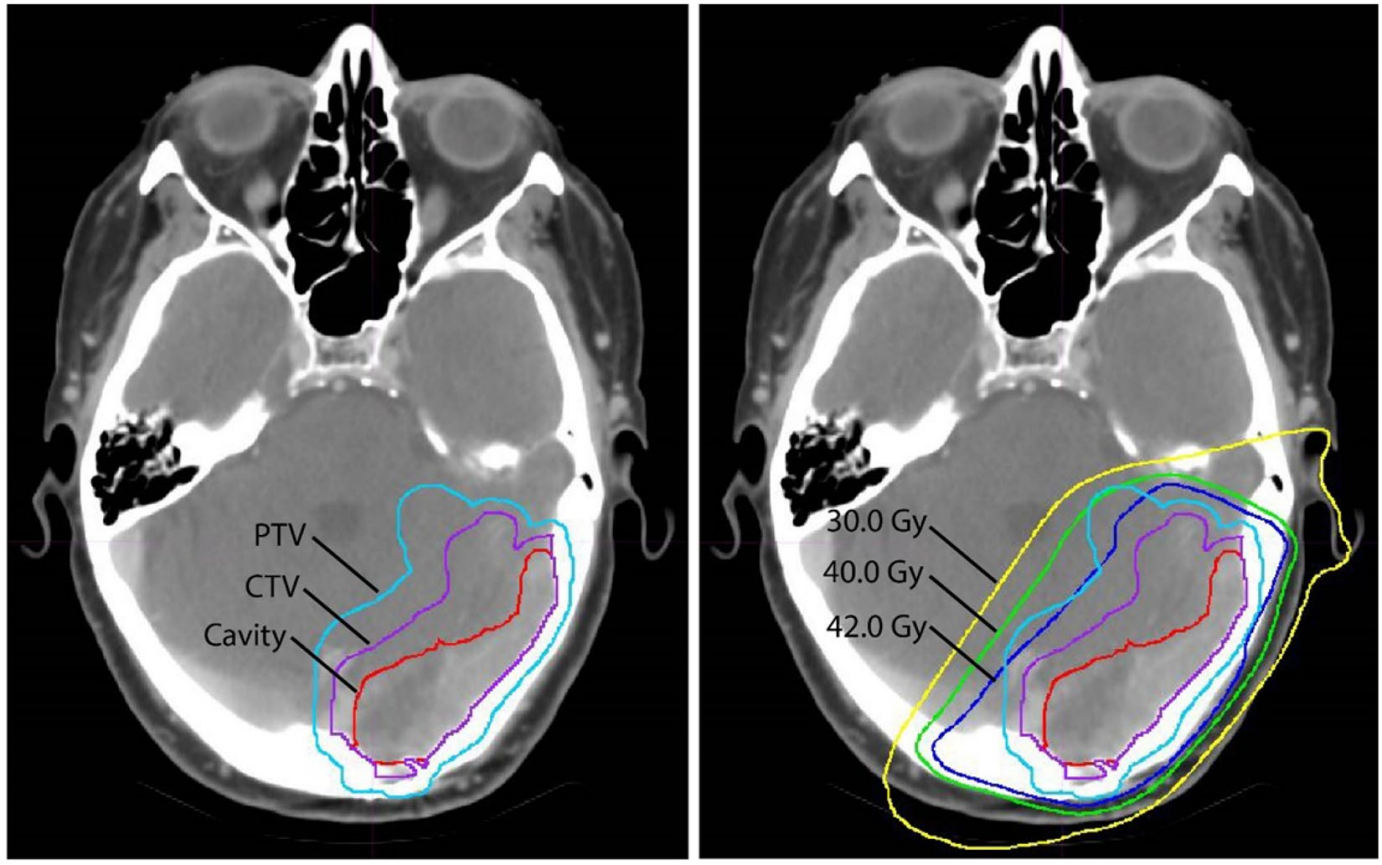
Example of (**A**) volumes and (**B**) dosimetric plan for a patient treated with post-operative cavity radiation therapy with standard fractionation.

Patients underwent immobilization with a 3-point thermoplastic mask. Target volumes were delineated using a CT obtained in the treatment position with registration to a contrast-enhanced diagnostic MRI. Both pre- and post-operative MRI brain scans were used for fusion and target definition. The surgical cavity was defined as the postoperative defect by post-contrast T1, surfaces original contacted by gross disease, and the surgical tract. The clinical target volume (CTV) was defined as the surgical cavity + 2 mm as well as up to 10 mm extension along regions of pre-operative dural contact but excluding anatomical barriers of spread such as bone. The planning target volume (PTV) was a uniform 3 mm expansion applied to the CTV (Fig. 1A,B). Volumetric analysis of the cavity and PTV were calculated using MIM (MIM Software Inc., Cleveland, OH). The number of treatment fractions varied between 10 and 14 based on histology. Higher total doses (39–42 Gy) were delivered to patients with historically radioresistant disease, such as melanoma, renal cell carcinoma, and colorectal cancer. Patients with more than 1 metastasis underwent fractionated radiation therapy to the surgical cavity and SRS to the additional lesions.

### Clinical endpoints

The following clinical endpoints were obtained from the electronic medical record, including time to local failure, distant brain failure, late radionecrosis, and overall survival. Local failure was defined as radiographic evidence of a new contrast-enhancing lesion, contiguous with or within the resection cavity. Pathological confirmation of local failure was not required for diagnosis. Distant brain failure was defined as development of new brain lesions separate from the surgical site; distant brain failure included diffuse leptomeningeal disease diagnosed by imaging results or examination of spinal fluid positive for malignant cells. Radionecrosis was based upon either pathologic confirmation or multidisciplinary consensus after radiology review. Time to local failure and distant brain failure was defined as the time from completion of treatment of the brain metastases to the MRI findings of disease.

### Statistical analyses

Actuarial rates were calculated by the Kaplan-Meier method. Competing risks analysis was performed using Stata (StataCorp., Stata Statistical Software: Release 15. College Station, TX: StataCorp LP). Variables evaluated for association with local failure included age, sex, Karnofsky performance status (KPS), extra-cranial metastases, more than 1 intra-cranial metastasis, histology, volume of cavity, volume of PTV, ratio of PTV/cavity and conformity index (CI).

### Ethical approval

All procedures performed in studies involving human participants were in accordance with the ethical standards of the institutional and/or national research committee and with the 1964 Helsinki declaration and its later amendments or comparable ethical standards.

### Informed consent

Informed consent was not obtained from individual participants included in this study due to the retrospective nature of this study.

## Results

### Clinical and treatment characteristics

A total of 45 patients were identified for analysis with a median follow-up of 7 months. Forty-four patients underwent photon radiation therapy, and 1 patient underwent proton radiation therapy. Four patients were lost to follow-up but were included in the analyses and censored at last follow-up. Table 1 summarizes the characteristics of the patient cohort. The median age at treatment was 66 years, and there were more men included in the study (27 compared to 18 women). The most common histologies included non-small cell lung cancer (NSCLC) (33%), melanoma (22%), and breast cancer (11%). Other histologies included endometrial, esophageal, ovarian, thyroid, and renal cell carcinoma. The median cavity volume was 20.5 cc (range 2.2–71.1 cc), and median PTV was 51.1 cc (range 7.3–176.4 cc). Furthermore, 15 patients (33%) received a CNS-active treatment (immune checkpoint inhibitor, BRAF inhibitor, or third-generation tyrosine kinase inhibitor) after radiation.

**Table 1:** Patient characteristics.

Characteristics	No.
Age	67 (37–88)
**Sex**	
Male	27
Female	18
KPS	80 (50–100)
**Extra-cranial metastases**	
Yes	24
No	21
Number of intracranial metastases	2 (1–9)
**Histology**	
NSCLC	15
Melanoma	10
Breast	5
Colorectal	2
Others	13
Median volume of cavity (cc)	20.5 (2.2–71.1)
Median volume of PTV (cc)	51.1 (7.3–176.4)
PTV/cavity volume	0.4 (0.1–0.6)
Conformity index	1.01 (0.1–2.0)

### Analysis of local failure, distant brain failure, and overall survival

**Figure 2:**
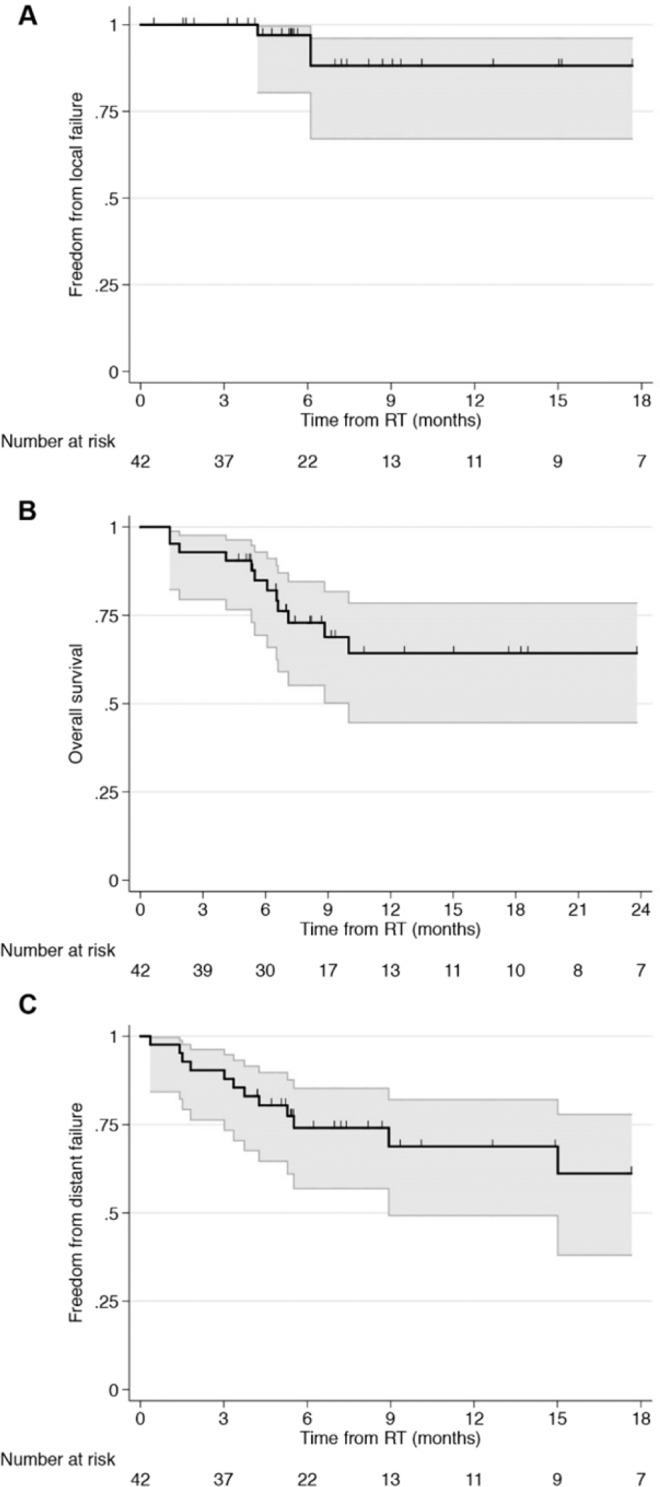
Kaplan-Meier curves for (**A**) freedom from local failure, (**B**) distant brain failure, and (**C**) overall survival.

The 6-month and 12-month freedom from local failure rates were 97.0% (95% CI, 80.4–99.6%) and 88.2% (95% CI, 67.1–96.1%) (Fig. 2A). Three patients (7%) experienced local failure at 4, 6, and 22 months. Two of the 3 patients were prescribed 30 Gy, and 1 patient was prescribed 39 Gy. All 3 failures were in-field. The histologies of the 3 patients who experienced local failure included colorectal adenocarcinoma (n = 1) and breast adenocarcinoma (n = 2). The cavity volumes of the 3 patients were 27.5, 24.6, and 8.3 cc. Of the patients who experienced local failure, 1 underwent stereotactic biopsy of the failure site, which confirmed recurrent adenocarcinoma. Two patients received SRS to the site of failure, and 1 patient did not pursue additional treatment. Of these two patients, SRS to the site of failure was effective in controlling the local disease. There were no events of radionecrosis appreciated by either consensus of imaging findings or biopsy confirmation including patients that underwent salvage therapy.

The 6-month and 12-month freedom from distant brain failure rates were 74.1% (95% CI, 56.9–85.3%) and 68.8% (95% CI, 49.2–82.1%), respectively (Fig. 2B). Nineteen patients (42%) experienced distant brain failure. The median time to distant brain failure was 15 months. On univariate analysis, there were no statistically significant predictors for distant brain failure (Table 2). Table 3 shows the percentage of patients by histology who experienced distant brain failure. Of the patients who experienced distant brain failure, 2 patients underwent resection of new distant brain metastasis followed by post-operative standard fractionation radiation therapy (not included in the analyses). For those who did not undergo surgical salvage, 2 patients were treated with standard fractionation radiation, 3 were treated with SRS to a single brain metastasis, and 1 patient was started on osimertinib for disease harboring an EGFR-mutation. Additional treatment was not pursued in 11 of the 19 patients.

**Table 2:** Univariate analysis for distant brain failure.

Characteristics	sHR	95% CI	P value
Age (>65)	2.06	0.70–6.04	0.2
**Sex**			
Male	1.68	0.56–5.07	0.4
KPS (> 80)	0.64	0.18–2.24	0.5
Extra-cranial metastases	2.22	0.79–6.24	0.1
**Number of intracranial metastases**			
>1 brain metastasis	0.78	0.46–1.34	0.4
**Histology**			
NSCLC	0.83	0.27–2.62	0.8
Melanoma	1.42	0.43–4.68	0.6
Cavity volume (>20 cc)	1.03	1.00–1.06	0.06
PTV volume (>50 cc)	1.00	0.99–1.02	0.6
PTV/cavity volume (>0.4)	3.81	0.02–871.89	0.6
CI (>1.0)	2.16	0.67–7.01	0.2

**Table 3:** Distant brain failure by histology.

Histologies	Number of patients (% of all patients with same histology)
NSCLC	5 (33%)
Melanoma	4 (40%)
Breast	4 (80%)
Colorectal	2 (100%)
Other	4 (31%)

The 6-month and 12-month overall survival rates were 84.9% (95% CI, 69.3–93.0%) and 64.3% (95% CI, 44.6–78.5%), respectively (Fig. 2C). There were 14 deaths in total. The median overall survival was 39 months. On univariate analysis, PTV volume >50cc was significantly associated with overall survival (Table 4).

**Table 4:** Univariate analysis for overall survival.

Characteristics	sHR	95% CI	P value
Age (>65)	1.96	0.60–6.39	0.3
**Sex**			
Male	1.05	0.36–3.06	0.9
KPS (>80)	0.21	0.04–1.09	0.06
Extra-cranial metastases	1.97	067–5.81	0.2
**Number of intracranial metastases**			
>1 brain metastasis	1.06	0.77–1.44	0.7
**Histology**			
NSCLC	1.38	0.45–4.23	0.6
Melanoma	1.71	0.57–5.15	0.3
Cavity volume (>20 cc)	1.02	0.98–1.06	0.3
PTV volume (>50 cc)	1.01	1.00–1.03	0.02
PTV/cavity volume (>0.4)	0.03	0.00–2.09	0.1
CI (>1.0)	2.39	0.58–9.83	0.2

## Discussion

There have been multiple prospective trials evaluating post-operative radiation therapy to reduce the risk of local failure for patients with brain metastases^4,6,7^. In this single-institution series, the use of postoperative radiation therapy with more standard fractionation (30–42 Gy in 3 Gy fractions), which was chosen to lower the biological effective dose (BED) to normal brain, widen the therapeutic index, and therefore afford the clinician the use of more generous treatment volumes. In this series, the 6-month and 12-month freedom from local failure rates were 97% and 88%, respectively and there were no events of late radionecrosis. These data compare favorably to the experiences of multiple prospective studies of SRS or SRT to the surgical cavity, which report 12-month local failure rates of 20–40% (Table 5)^4,6,7,9^.

**Table 5:** Summary of results of post-operative cavity radiation therapy.

Modality	Number of patients	12-month freedom from LF	12-month freedom from DF	Median OS (months)	Ref.
SRS	64	72%	42%	17	(6)
SRS	98	62%	65%	12.2	(7)
WBRT	96	87%	89%	11.6	(7)
SRT	20	79%	63%	23.6	(9)
WBRT	49	90%*	86%*	11.1	(4)

LF – local failure, DF – distant failure; *Overall freedom from LF or DF.

Local control in the postoperative setting may be improved with the use of generous treatment volumes. It should be noted that the risk of leptomeningeal disease after cavity SRS has been found to be between 8–25%, many of which are locoregional failures^18–21^. Early data from Stanford demonstrated that lower conformality was associated with improved local control in the use of single-fraction SRS for treatment of postoperative cavities^11^. Taken together, it is plausible that the high risk of local failure after single-fraction SRS might be explained by tight treatment volumes that do not fully encompass the tumor bed margins at risk of residual microscopic disease. A recently published guideline on contouring postoperative cavities advocates for inclusion of the entire surgical tract and 5–10 mm extension along regions of pre-operative dural contact^10^. The use of fractionation may afford the clinician the confidence to safely use generous treatment volumes. For example, in this study, the median volume of the contoured surgical cavity was 20.5 cc compared to 8.9 cc reported in a prospective trial of postoperative SRS^8^. In addition, at our institution, we favor a more protracted radiation schedule with lower dose per fraction given the expected low α/β of normal brain tissue. For example, in a recently published series the risk of adverse radiation effects from a median of 30 Gy in 5 fractions is > 20%^22^. This population does not different from those larger populations.

Late radionecrosis after radiation therapy is a growing concern as patients with brain metastases are living longer due to the availability of systemic agents with CNS penetrance. In this setting, the neurocognitive effects of radionecrosis may deprive patients of quality of life to an extent that rivals tumor progression. A recent retrospective study found after cavity SRS a 1-year rate of symptomatic radionecrosis of 6.0% for patients treated with a margin of <1.0 mm and up to 20.9% for patients with a margin of >1.0 mm^23^. In our series, there were no observed incidents of late radionecrosis, and this is likely related to the lower biological effective dose to normal tissues. The equivalent BED in 2 Gy fractions assuming an α/β of 2 for normal brain (i.e., BED2/2) ranges from 37.5–52.5 Gy using a dose and fractionation for 30–42 Gy in 3 Gy fractions. This BED2/2 would be expected to produce very low risk of late radionecrosis as it is lower than most doses used in partial brain irradiation for primary brain tumors. Furthermore, this fractionated radiation course has a lower BED than SRS.

Postoperative radiation therapy delivered with more standard fractionation offers multiple logistical benefits. Daily physician and stereotactic physics support is not required for treatment delivery. This approach may also conserve on allocation of resources including immobilization devices, stereotactic image-guidance, and overuse of intensity modulated radiation therapy. In the follow-up clinic, this fractionation nearly eliminates the common dilemma of having to differentiating tumor recurrence from late radionecrosis. Therefore, the clinician may make earlier decisions about the need for salvage therapy as opposed to continuing surveillance.

This study has several limitations. Although our patients are followed routinely with imaging and clinical visits ever 2–3 months, this was not a prospective study. Prospective patient reported quality of life data was not reported. Lastly, in this era of systemic agents with CNS activity, long-term follow-up for intracranial control and overall survival will require the maturation of data for years to come.

In summary, post-operative radiation therapy to the surgical cavity with standard fractionation results in very low risks of local failure and late radionecrosis. This may represent an effective, safe, and straightforward alternative to postoperative SRS, SRT, and WBRT. The results of this study warrant a randomized prospective trial comparing single-fraction SRS to more standard fractionation therapy in the postoperative management of brain metastases.
